# Intracortical microstructure profiling: A cross-modal method for indexing cortical lamination

**DOI:** 10.1162/IMAG.a.1212

**Published:** 2026-04-21

**Authors:** Casey Paquola, Jessica Royer, Thanos Tsigaras, Donna Gift Cabalo, Youngeun Hwang, Felix Hoffstaedter, Simon B. Eickhoff, Boris C. Bernhardt

**Affiliations:** Institute for Neuroscience and Medicine (INM-7), Forschungszentrum Juelich, Juelich, Germany; McConnell Brain Imaging Centre (BIC) and Centre for Excellence in Epilepsy at the Neuro (CEEN), Montreal Neurological Institute, McGill University, Montreal, Canada; Max Planck Institute for Human Cognitive and Brain Sciences, Leipzig, Germany; Institute for Systems Neuroscience, Heinrich Heine Universität Dusseldorf, Dusseldorf, Germany

**Keywords:** quantitative MRI, cytoarchitecture, myeloarchitecture, in vivo histology, surface-based analysis, open-source pipeline

## Abstract

Intracortical microstructure profiling represents a powerful, scalable approach for investigating the laminar organisation of the human cortex on both *in-vivo* and *post-mortem* datasets. Building upon a long tradition of histological analysis, this method leverages surface-based intracortical sampling to generate profiles of tissue properties across cortical depths. The present work outlines a standardised workflow for intracortical microstructural profiling, newly packaged as the open-source toolbox “CortPro” (https://github.com/caseypaquola/cortpro). Here, we explore the utility of central moments as descriptors of profile shape. Using these measures, we quantify *(i)* the extent to which *in-vivo* MRI can capture laminar differentiation, *(ii)* the test-retest reliability of profiles, and *(iii)* their replicability across sites and studies. Our results demonstrate that intracortical profiles are remarkably robust and effectively mitigate bias-field related limitations of non-quantitative MRI. As applications of microstructure-sensitive imaging expand across development, aging, and disease, microstructure profiling provides a principled means of linking microstructural neuroanatomy with systems-level brain organisation.

## 1 Background and Introduction

Neuronal morphology, density and myelination vary across cortical depths (Nieuwenhuys, 2013; Von Economo & Koskinas, 1925). While this stratification is fundamental to contemporary theories of brain function (Douglas et al., 1989; Felleman & Van Essen, 1991), research into its variability across time, space and individuals has been hampered by the challenge of systematically probing intracortical architecture.

Parallel advances in histology and neuroimaging have recently reinvigorated research into intracortical architecture. On the one hand, new alignment techniques enabled reconstruction of histological sections into 3D volumes (Alkemade et al., 2022; Amunts et al., 2013), thereby allowing cortical layers to be visualised across the entire human brain regardless of the cutting plane (Novek et al., 2023). On the other hand, sub-millimetre, microstructure-sensitive magnetic resonance imaging (MRI) has recently been made feasible, owing to higher magnetic field strengths, acceleration factors and sequencing innovations (Bock et al., 2013; Cabalo et al., 2025; Dinse et al., 2015; Royer et al., 2022; Weiskopf et al., 2021). The range of intracortical microstructural properties accessible with *in-vivo* imaging is also expanding, from myelin and iron to astrogliosis and vasculature (Benjamini et al., 2023; Gulban et al., 2026; Stüber et al., 2014).

Capitalising on these innovations, we developed a standardised workflow for *intracortical profiling*, which charts variations in signal from the pial mater to the grey/white matter boundary. Intracortical profiling was first developed for 2D micrographs (Braitenberg, 1962), and inspired various instantiations of layer-wise analysis of myelin-sensitive and functional MRI (Dinse et al., 2015; Koopmans et al., 2010; Polimeni et al., 2010). Here, we specifically aim to highlight how intracortical microstructure profiling enables objective characterisation, presenting a promising way forward to understanding differences across regions and individuals.

The implementation of intracortical profiling *in-vivo* has launched a bountiful new era of investigations into cortical microstructure. Recently, researchers have identified differences in cortical microstructure related to biological (Küchenhoff et al., 2024; Park et al., 2024; Royer et al., 2023; Sprooten et al., 2019; Wei et al., 2022) and cognitive-behavioural factors (Lee et al., 2025; Patel et al., 2023; Valk et al., 2023), including particularly strong changes with developmental and lifespan processes (Hettwer et al., 2024; Paquola, Bethlehem, et al., 2019; Sui et al., 2022; Sydnor et al., 2025; Whitaker et al., 2016). More generally, microstructure profiles enabled the first quantitative evidence that the long-discussed sensory-fugal axis predominates cortical organisation (Mesulam, 1998; Paquola, Vos De Wael, et al., 2019; Sanides, 1962). Furthermore, as the sensory-fugal axis reflects differences in supra- vs. infra-granular microstructure, positioning activation patterns or group differences on this axis has helped to shed light on their position in cortical hierarchies.

While this field is still in its infancy, *in-vivo* profiling alongside fMRI avails a huge realm of possibilities for uncovering the dependences between microstructure and function in the human brain and potentially unravelling mechanisms of cognition (Paquola et al., 2022). This approach offers exciting synergies with laminar fMRI analysis, which has already shown the importance of layer-wise interactions for understanding cortical organisation and dynamics (Finn et al., 2019; Huber et al., 2017).

## 2 The Intracortical Microstructure Profiling Workflow

A central methodological consideration in intracortical profiling concerns how sampling points are distributed across cortical depths. Cortical layers vary in thickness as a function of cortical curvature, with upper layers tending to be thicker in sulci and deeper layers thicker in gyri (Bok, 1929). To account for this, sampling points can be positioned using an equivolumetric algorithm, which maintains a constant volume fraction for each cortical segment (Waehnert et al., 2014). In essence, this approach ensures that a given fraction of cortical volume is represented equally across regions, much like how the top centimetre of a wide martini glass can contain the same amount of liquid as several centimetres near its narrow base. Operationally, equivolumetric surface generation involves adjusting the depth of each vertex on the intracortical surface mesh according to its position within sulci or gyri. This yields surfaces that more closely follow true cortical lamination compared with alternative approaches such as equidistant or Laplacian-based sampling (Waehnert et al., 2014). A note of caution, however, inaccuracies in cortical surface segmentation partially propagate through the equivolumetric layering, making it more sensitive to imprecision than equidistant models (Gülban & Huber, 2025). In general, the accuracy of the cortical segmentation is of paramount importance to achieving anatomically-realistic measures of the intracortical microstructure. Furthermore, layer thickness varies between cortical areas (Von Economo & Koskinas, 1925). Consequently, equivolumetric sampling preserves relative layering within a given area, but the same fractional depth may not correspond to an identical histological layer across areas. Intracortical microstructure profiling is, therefore, sensitive to patterns of lamination rather than absolute differences within specific layers. This representation nonetheless provides valuable insight into regional microarchitecture and fine-grained cortical differentiation (García-Cabezas et al., 2020; Paquola et al., 2025).

Secondly, the appropriate density of sampling is dependent on the resolution of the underlying image. Take, for example, an MRI of an adult brain with isotropic resolution of 0.8 mm. Given an average cortical thickness of 2.8 mm, one may assume that a profile captures approximately 3.2 unique voxels. In practice, the number is significantly higher, because cortical surfaces are variably positioned relative to voxels (mean ± SD: 5.6 ± 1.5 voxels, Supplementary Fig. S1). Additionally, this calculation assumes nearest-neighbour interpolation, but trilinear interpolation is typically advised to avoid stair-step artefacts. Trilinear interpolation involves taking a weighted average of eight neighbouring voxels for each sampling point, thereby producing a smooth estimate of continuous transitions in image intensities. Using this technique, the number of unique values per profile drastically increases (mean ± SD: 38.7 ± 6.4). In our standard workflow, we recommend striking a balance between these nearest neighbour and trilinear estimates of unique values (e.g.*,* 14 samples), thereby limiting redundancy in the profile while still capturing a full spectrum of microstructural variations across cortical depths. Another note of caution, however, the relative imaging resolution still differs depending on regional cortical thickness (Gülban & Huber, 2025), and it is advisable to test that regional differences in profile properties are maintained after controlling for this.

**Fig. 1.**
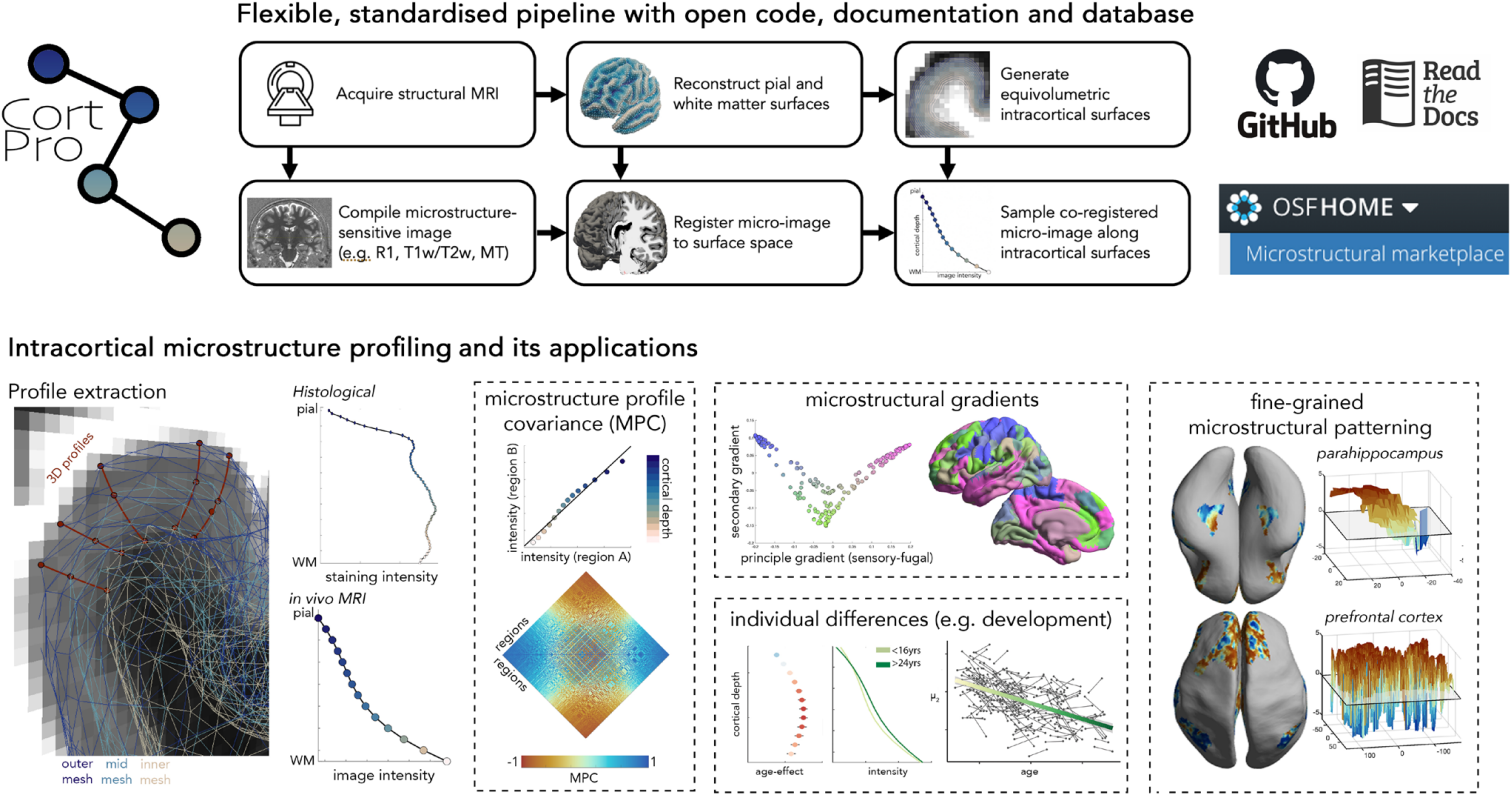
(Above) Flowchart of intracortical microstructure profiling approach. The acquisition protocol should include T1-weighted imaging (e.g., MPRAGE) and a high-resolution (≤1mm) sequence that enables a microstructural contrast (e.g. MP2RAGE (Marques et al., 2010), SPACE for T2-weighted (Mugler et al., 2000) or MPM (Weiskopf et al., 2013)). The T1w image is optimal for most cortical surface reconstruction pipelines (e.g., Freesurfer), while a microstructure-sensitive image (‘micro-image’) may be compiled in a variety of ways (e.g., R1 from MP2RAGE or MPM, or computation of T1w/T2w). The micro-image is then rigidly aligned to the surface-space T1w image. Finally, following generation of equivolumetric surfaces between the pial and GM/WM boundary, the intensities of the co-registered micro-image are sampled along the intracortical surfaces at matched vertices to produce microstructure profiles. (Below) 3D profile extraction is operationalised by sampling matched vertices on intracortical surfaces of various cortical depths. The protocol provides a consistent approach that can be used on different modalities, such as histology and MRI. Resulting profiles can be explored in a wide variety of ways, including computation of a matrix of regional similarities in microstructure (i.e., microstructure profile covariance) and gradients of microstructural differentiation (Paquola, Vos De Wael, et al., 2019); individual differences in microstructure (e.g.*,* during development (Paquola, Bethlehem, et al., 2019)) or fine-grained microstructural patterning with regions (Paquola et al., 2025).

Building on these insights, we developed a standardised workflow for extracting microstructure profiles (MPs), using a combination of commands from FreeSurfer (Fischl, 2012), FSL (Jenkinson et al., 2012) and AFNI (Cox, 1996). The procedure involves: *(i)* reconstructing the cortical surface, *(ii)* generating equivolumetric intracortical surfaces with matched vertex indices, *(iii)* registering a microstructure-sensitive image to surface space, (iv) sampling voxel intensities along the matched intracortical surfaces, and (v) compiling these intensity values into profiles that capture depth-wise variations in tissue properties (Fig. 1, see Supplementary Material for full protocol).

The workflow is now available as a standalone toolbox (https://github.com/caseypaquola/cortpro) and incorporated within the open access multimodal MRI processing software “micapipe” (Cruces et al., 2022). The toolbox works flexibly on MRI and 3D histology, given a cortical surface reconstruction (pial and white matter boundary) and an aligned volume for sampling. To support integration with existing workflows and common imaging sequences, we have incorporated several optional preprocessing steps into the CortPro toolbox, including cortical surface reconstruction using FastSurfer (Henschel et al., 2020) and computation of an N3 bias-corrected T1w/T2w ratio image (Nerland et al., 2021). Therefore, the minimal requirements for initiating the CortPro workflow are a quantitative T1, or a T1w and T2w image (Fig. 1 top middle).

**Fig. 2.**
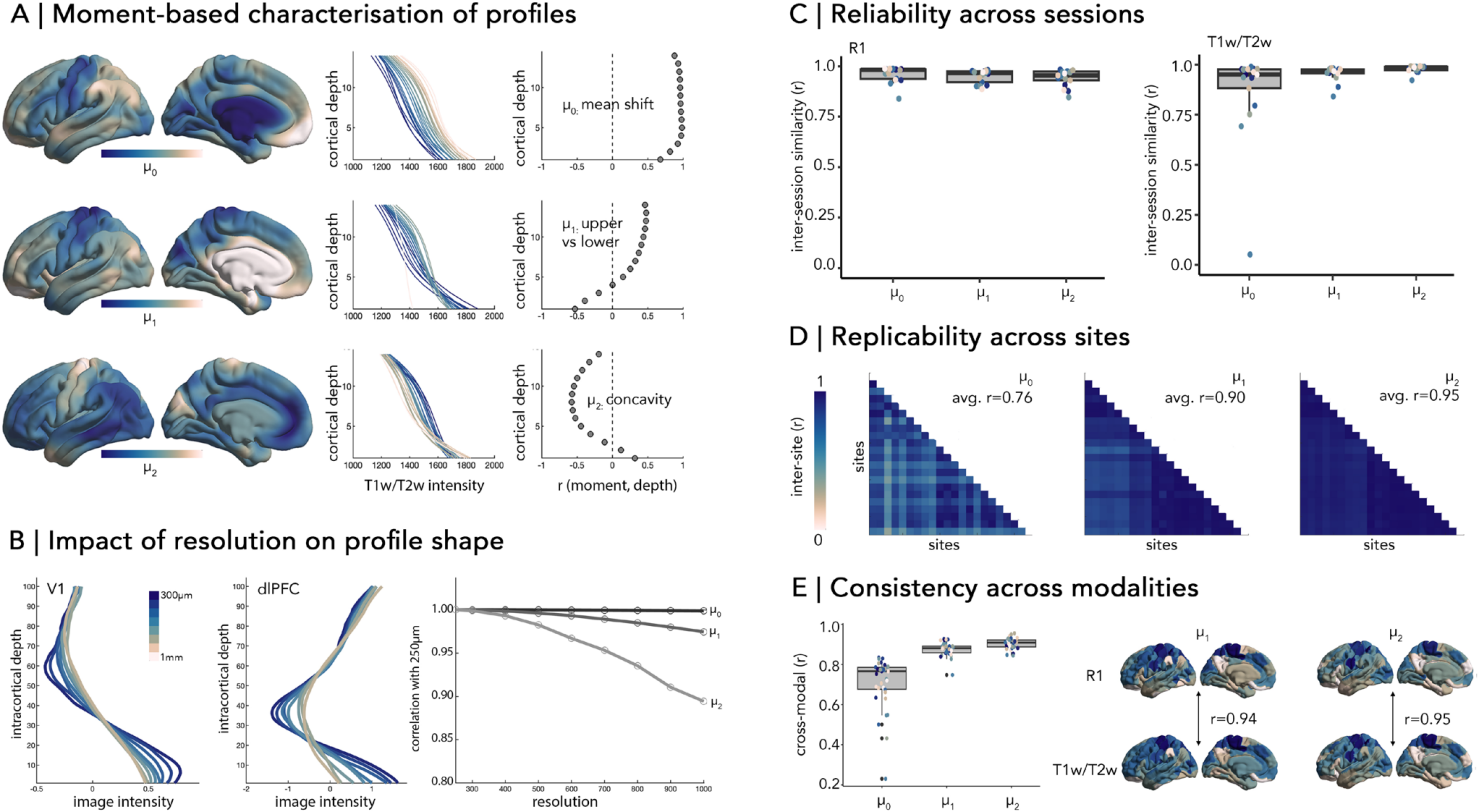
(A) *(Left)* Regional variation in zeroth to second moment [R1 at 3T, (Royer et al., 2022)]. *(Middle)* Line plots represent average profiles within bins of the moment spectrum, with colours matched to the cortical plots, illustrating how the moments are related to change in profile shapes. *(Right)* Spatial correlation of moments with depth-wise intensities show differential sensitivities to shape. (B) *(Left)* Intracortical profiles derived from increasingly downsampled structural MRI (original acquisition = 0.25 mm) acquired in one individual (Lüsebrink et al., 2021). *(Right)* Line plots show the similarity of cortex-wide moment maps computed on downsampled volumes, relative to the original ultra-high resolution volume (0.25 mm). (C) Boxplots show inter-session stability of profile moments, with points coloured by subject. (D) Matrices illustrate the correlations between group-average moment maps derived from T1w/T2w imaging, acquired at 21 different sites. (E) Boxplots show the within-subject correlations of moment maps derived from different modalities (R1 and T1w/T2w). Cortical surfaces depict group-average maps of μ1 and μ2 derived from R1 (*above*) and T1w/T2w (*below*) in the same sample. Note: Further details on these analyses can be found in the Supplementary Material.

Comparing MPs across areas, individuals or groups is simplified by using their central moments (Fig. 2A). The zeroth moment (μ0) is the mean intensity and thus disregards variation in intensities across cortical depths. The higher moments (μ1-μ4) are, in contrast, sensitive to profile shape. The centre of gravity (μ1) indicates the balance of intensities in supra-vs-infragranular layers, while variance (μ2) reflects the spread of intensities across depths. For MRI-derived profiles, μ2 effectively indexes the flatness of the profile. Finally, skewness (μ3) and kurtosis (μ4) capture intensity shifts in the tails of the profiles (i.e.*,* uppermost and lowermost depths) and for imaging data tend to be highly correlated with μ2 and μ1, respectively (|r| > 0.92, Supplementary Fig. S1). Notably, the zeroth to second moments capture distinct patterns of microarchitectural variation (spatial correlation between moments: 0.34 < |r| < 0.60, Fig. 2A). Therefore, moment maps offer complementary information on cortical differentiation. For *in-vivo* imaging, the central moments offer sufficient granularity and distinctiveness to describe the smooth profiles, but with higher resolution data more elaborate features may be appropriate.

## 3 Proxying Cortical Lamination with MRI

Standard microstructure-sensitive imaging sequences are now able to reach sub-millimetre resolution with relatively short scan times but identifying cortical laminar or prominent myelin bands (e.g., stria of Gennari) depends upon even higher resolution imaging (i.e., < 0.5 mm). Several studies have achieved this goal with *in-vivo* MRI, but they require long scan times or small fields of view that are typically infeasible to collect in large-scale or clinical studies. Therefore, an important assumption of the present approach is that the shape of the microstructure profile can still be resolved up to a certain degree at lower resolutions.

To test this point, we compared microstructure profiles and moment maps at a range of resolutions, based on the downsampling of a T1w image acquired at 0.25 mm resolution in one individual (Lüsebrink et al., 2021). While the profiles are clearly smoother at lower resolutions, the moment maps are strikingly similar even at 1 mm resolution (r > 0.9) (Fig. 2B). At 0.7 mm resolution, the correlations even exceed 0.95. Higher moments are increasingly sensitive to resolution (r_1mm_ μ0 = 1.0, μ1 = 0.98, μ2 = 0.90), which reinforces a selective focus on μ0-μ2 in *in-vivo* imaging studies. In sum, microstructure profiling with 0.7 - 1 mm resolution imaging can still capture the regional organisation of cortical laminar.

## 4 Reliability and Replicability of MPs

As with any method, the utility of intracortical microstructure profiling in research and clinical studies is dependent upon reliability and replicability. To assess these attributes, we analysed several open-access datasets that involved multi-session, multi-modal imaging (Cabalo et al., 2025; Royer et al., 2022; Shams et al., 2019), or acquisition across sites with distinct scanning protocols (Casey et al., 2018). Comprehensive descriptions of these datasets and preprocessing are detailed in the Supplementary Material (Supplementary Tables S1–S3).

Test-retest (or scan-rescan) reliability provides insight into measurement error and is especially important for longitudinal studies that aim to capture meaningful intra-individual variation. To assess reliability, we compared individual-specific moment maps derived from different scanning sessions [n = 17, 2 sessions within 1 day (Shams et al., 2019)]. The scanning protocols involved both quantitative T1 mapping (longitudinal relaxation time, “R1”) and non-quantitative acquisitions of T1w and T2w contrasts. Moment maps were very highly correlated across sessions for both R1 (r = 0.95 ± 0.04) and T1w/T2w (r = 0.95 ± 0.11) (Fig. 2C), with the exception of μ0 in T1w/T2w imaging (r = 0.87 ± 0.23). This effect is likely related to B1+ transmit field biases that are known to influence T1w/T2w intensities (Glasser et al., 2022). Higher moments (μ1-μ4) sidestep this issue as their computation depends on relative values within an area, thus being relatively resilient to low spatial frequency variations that are characteristic of the B1+ field. Corroborating these results, we also found that the intraclass correlation coefficient (ICC), which is more sensitive to the discriminability of individuals, ranged from moderate to high across moments and regions, with the exception of low values for μ0 in T1w/T2w imaging [R1: ICC(3,1) = 0.70 ± 0.19, T1w/T2w: ICC(3,1) = 0.70 ± 0.30, Supplementary Fig. S2]. Notably, these ICC values position the reliability of microstructure profiles on par with structural volumetry (Morey et al., 2010) and cortical thickness (Iscan et al., 2015) and superior to functional MRI (Elliott et al., 2020). We found similarly high levels of reliability in two additional datasets that involved longer durations between scans [PNI: n = 10, inter-session interval = 1.9 ± 2.5 months, inter-session similarity = 0.95 ± 0.04 (Cabalo et al., 2025). MICs: n = 40, inter-session interval = 1.71 ± 1.03 years, inter-session similarity = 0.89 ± 0.05 (Royer et al., 2022), Supplementary Fig. S2]. Thus, the shape of microstructure profiles, as captured by μ1-μ4, is highly reliable, even if the reliability of the regional intensity values is not, as is the case for T1w/T2w.

Replicability reflects the independence of imaging features to site-specific effects. With global data aggregation necessary to grasp population diversity, ensuring the consistency of imaging features across sites is increasingly important. Thus, we examined the similarity of moment maps acquired across 21 sites, including 3 different scanner manufacturers [ABCD dataset; (Casey et al., 2018), Supplementary Fig. S3]. Given the substantial sample size (n = 5046) and the consistency of age distributions across sites (Supplementary Fig. S3), we expected that any systematic differences in moment maps would primarily reflect scanner-related factors. Inter-site replicability of moment maps (product-moment correlation across sites) was very high, especially for μ2 (r = 0.95 ± 0.03) and μ1 (r = 0.90 ± 0.08) (Fig. 2D). Replicability was lower for μ0 (r = 0.76 ± 0.13), which as above may be attributed to the influence of B1 transmit field bias. Together, these findings highlight the robustness of intracortical microstructure profiling in producing replicable metrics of cortical architecture that generalise across diverse scanner models and acquisition environments.

Finally, we evaluated whether intracortical microstructure profiles can be compared across modalities. Specifically, we assessed the similarity of moment maps derived from different myelin-sensitive contrasts (R1 and T1w/T2w) that were acquired within the same session for 17 individuals (Shams et al., 2019). At an individual level, cross-modal correlation of moment maps was very high for μ2 (r = 0.90 ± 0.03) and μ1 (r = 0.87 ± 0.03) (Fig. 2E). In line with previous analyses, the consistency of μ0 was lower (r = 0.71 ± 0.10). We found a similar pattern of results with comparison of more distinct myelin-sensitive sequences [R1 and MTsat, (Cabalo et al., 2025)], whereby μ2 (r = 0.47 ± 0.12) and μ1 (r = 0.40 ± 0.10) were more similar across modalities than μ0 (r = 0.16 ± 0.13) (Supplementary Fig. S4). Thus, examination of the shape of the profile can help to overcome idiosyncrasies of specific modalities and potentially bridge across datasets that use different sequences.

## 5 Conclusion

With microstructure-sensitive imaging entering the mainstream of neuroimaging research, profiling offers a versatile and reliable approach for indexing cortical lamination. The present findings indicate that the shape of intracortical intensity profiles (as described by μ1 and μ2) offers a reliable and replicable basis for assessing inter-individual differences in cortical microstructure. Future studies would benefit from incorporating quantitative relaxometry (*e.g.,* R1 mapping) to facilitate region-wise comparisons of microstructural properties, as reliability and replicability decline when using non-quantitative contrasts such as T1w/T2w ratios. More broadly, our results suggest that 1 mm isotropic MRI is sufficient to capture meaningful laminar variation, though higher resolutions can provide measurable improvements in sensitivity and precision, and are becoming increasingly attainable with contemporary acquisition protocols (e.g., 0.65 mm, MP2RAGE at 3T within ~9 min (Bapst et al., 2024)).

The robustness and reliability of intracortical microstructural markers support their application in large-scale neuroimaging studies and biomedical research. To support future applications of the intracortical microstructure profiling approach, we developed a generalisable workflow for their extraction (https://github.com/caseypaquola/cortpro) and incorporated the code into an automated data preprocessing toolbox [micapipe, (Cruces et al., 2022)]. The modular nature of the workflow is well-poised to adapt to advances in the field, such as improvements to layer segmentation, and new options are being readily integrated via GitHub issues. Furthermore, we are releasing a warehouse of microstructure profiles and moment maps derived from open-access resources (“Microstructural Marketplace”, https://osf.io/e6f7d/). These include a wide range of microstructure-sensitive contrasts (R1, T1w/T2w, MTsat) acquired at various resolutions (0.25 mm–0.8 mm) and different magnetic strengths (3T and 7T) (Cabalo et al., 2025; Lüsebrink et al., 2021; Royer et al., 2022; Shams et al., 2019), as well as post-mortem staining for cells bodies [BigBrain: (Amunts et al., 2013; Paquola et al., 2021)], myelin and parvalbumin interneurons [AHEAD: (Alkemade et al., 2022)]. We hope these datasets provide a helpful benchmark for new profiling studies.

Microarchitecture occupies an intriguing middle ground between the relatively stable genetic code and highly dynamic neural activity. It emerges from intrinsic developmental programs and constrains cortical function, while also being continuously shaped by experience-dependent plasticity. Understanding these complex, reciprocal interactions requires robust *in vivo* imaging approaches that enable direct integration of multiple modalities within individuals. Looking ahead, integrating microstructural profiling with depth-resolved functional and vasculature imaging offers an exciting path towards better understanding of information processing in cortical microcircuits.

## Supplementary Material

Supplementary Material

## Data Availability

Raw data for our analyses are available via the following open datasets: Lüsebrink: https://open-science.ub.ovgu.de/items/5d8e3df2-f60f-44a9-b2c7-710fc1a142a0/full Shams: https://data.donders.ru.nl/collections/di/dccn/DSC_3015046.03_479?26 MICA-MICs: https://osf.io/j532r/ MICA-PNI: https://osf.io/mhq3f/ ABCD: https://www.nbdc-datahub.org/ Preprocessed microstructure profiles are additionally available on the Microstructure Marketplace (https://osf.io/e6f7d/overview). Code used to conduct the analyses are available as part of CortPro (https://github.com/caseypaquola/CortPro).

## References

[IMAG.a.1212-b1] Alkemade, A., Bazin, P.-L., Balesar, R., Pine, K., Kirilina, E., Möller, H. E., Trampel, R., Kros, J. M., Keuken, M. C., Bleys, R. L. A. W., Swaab, D. F., Herrler, A., Weiskopf, N., & Forstmann, B. U. (2022). A unified 3D map of microscopic architecture and MRI of the human brain. Science Advances, 8(17), eabj7892. 10.1126/sciadv.abj7892 35476433 PMC9045605

[IMAG.a.1212-b2] Amunts, K., Lepage, C., Borgeat, L., Mohlberg, H., Dickscheid, T., Rousseau, M.-E., Bludau, S., Bazin, P.-L., Lewis, L. B., Oros-Peusquens, A.-M., Shah, N. J., Lippert, T., Zilles, K., & Evans, A. C. (2013). BigBrain: An ultrahigh-resolution 3D human brain model. Science, 340(6139), 1472–1475. 10.1126/science.1235381 23788795

[IMAG.a.1212-b4] Bapst, B., Massire, A., Mauconduit, F., Gras, V., Boulant, N., Dufour, J., Bodini, B., Stankoff, B., Luciani, A., & Vignaud, A. (2024). Pushing MP2RAGE boundaries: Ultimate time-efficient parameterization combined with exhaustive T1 synthetic contrasts. Magnetic Resonance in Medicine, 91(4), 1608–1624. 10.1002/mrm.29948 38102807

[IMAG.a.1212-b5] Benjamini, D., Priemer, D. S., Perl, D. P., Brody, D. L., & Basser, P. J. (2023). Mapping astrogliosis in the individual human brain using multidimensional MRI. Brain, 146(3), 1212–1226. 10.1093/brain/awac298 35953450 PMC9976979

[IMAG.a.1212-b7] Bock, N. A., Hashim, E., Janik, R., Konyer, N. B., Weiss, M., Stanisz, G. J., Turner, R., & Geyer, S. (2013). Optimizing T1-weighted imaging of cortical myelin content at 3.0T. NeuroImage, 65, 1–12. 10.1016/j.neuroimage.2012.09.051 23036446

[IMAG.a.1212-b8] Bok, S. T. (1929). Der Einfluß der in den Furchen und Windungen auftretenden Krümmungen der Großhirnrinde auf die Rindenarchitektur. Zeitschrift Für Die Gesamte Neurologie Und Psychiatrie, 121(1), 682–750. 10.1007/BF02864437

[IMAG.a.1212-b9] Braitenberg, V. (1962). A note on myeloarchitectonics. The Journal of Comparative Neurology, 118(2), 141–156. 10.1002/cne.901180202 13872421

[IMAG.a.1212-b10] Cabalo, D. G., Leppert, I. R., Thevakumaran, R., DeKraker, J., Hwang, Y., Royer, J., Kebets, V., Tavakol, S., Wang, Y., Zhou, Y., Benkarim, O., Eichert, N., Paquola, C., Doyon, J., Tardif, C. L., Rudko, D., Smallwood, J., Rodriguez-Cruces, R., & Bernhardt, B. C. (2025). Multimodal precision MRI of the individual human brain at ultra-high fields. Scientific Data, 12, 526. 10.1038/s41597-025-04863-7 40157934 PMC11954990

[IMAG.a.1212-b11] Casey, B. J., Cannonier, T., Conley, M. I., Cohen, A. O., Barch, D. M., Heitzeg, M. M., Soules, M. E., Teslovich, T., Dellarco, D. V., Garavan, H., Orr, C. A., Wager, T. D., Banich, M. T., Speer, N. K., Sutherland, M. T., Riedel, M. C., Dick, A. S., Bjork, J. M., Thomas, K. M., … Dale, A. M. (2018). The Adolescent Brain Cognitive Development (ABCD) study: Imaging acquisition across 21 sites. Developmental Cognitive Neuroscience, 32, 43–54. 10.1016/j.dcn.2018.03.001 29567376 PMC5999559

[IMAG.a.1212-b12] Cox, R. W. (1996). AFNI: Software for analysis and visualization of functional magnetic resonance neuroimages. Computers and Biomedical Research, 29(3), 162–173. 10.1006/cbmr.1996.0014 8812068

[IMAG.a.1212-b13] Cruces, R. R., Royer, J., Herholz, P., Larivière, S., Vos de Wael, R., Paquola, C., Benkarim, O., Park, B.-Y., Degré-Pelletier, J., Nelson, M. C., DeKraker, J., Leppert, I. R., Tardif, C., Poline, J.-B., Concha, L., & Bernhardt, B. C. (2022). Micapipe: A pipeline for multimodal neuroimaging and connectome analysis. NeuroImage, 263, 119612. 10.1016/j.neuroimage.2022.119612 36070839 PMC10697132

[IMAG.a.1212-b14] Dinse, J., Härtwich, N., Waehnert, M. D., Tardif, C. L., Schäfer, A., Geyer, S., Preim, B., Turner, R., & Bazin, P.-L. (2015). A cytoarchitecture-driven myelin model reveals area-specific signatures in human primary and secondary areas using ultra-high resolution in-vivo brain MRI. NeuroImage, 114, 71–87. 10.1016/J.NEUROIMAGE.2015.04.023 25896931

[IMAG.a.1212-b15] Douglas, R. J., Martin, K. A. C., & Whitteridge, D. (1989). A canonical microcircuit for neocortex. Neural Computation, 1(4), 480–488. 10.1162/neco.1989.1.4.480

[IMAG.a.1212-b16] Elliott, M. L., Knodt, A. R., Ireland, D., Morris, M. L., Poulton, R., Ramrakha, S., Sison, M. L., Moffitt, T. E., Caspi, A., & Hariri, A. R. (2020). What is the test-retest reliability of common task-functional MRI measures? New empirical evidence and a meta-analysis. Psychological Science, 31(7), 792–806. 10.1177/0956797620916786 32489141 PMC7370246

[IMAG.a.1212-b17] Felleman, D. J., & Van Essen, D. C. (1991). Distributed hierarchical processing in the primate cerebral cortex. Cerebral Cortex, 1(1), 1–47. 10.1093/cercor/1.1.1-a 1822724

[IMAG.a.1212-b18] Finn, E. S., Huber, L., Jangraw, D. C., Molfese, P. J., & Bandettini, P. A. (2019). Layer-dependent activity in human prefrontal cortex during working memory. Nature Neuroscience, 22(10), 1687–1695. 10.1038/s41593-019-0487-z 31551596 PMC6764601

[IMAG.a.1212-b19] Fischl, B. (2012). FreeSurfer. Neuroimage, 62(2), 774–781. 10.1016/j.neuroimage.2012.01.021 22248573 PMC3685476

[IMAG.a.1212-b20] García-Cabezas, M. Á., Hacker, J. L., & Zikopoulos, B. (2020). A protocol for cortical type analysis of the human neocortex applied on histological samples, the atlas of Von Economo and Koskinas, and magnetic resonance imaging. Frontiers in Neuroanatomy, 14, 576015. 10.3389/fnana.2020.576015 33364924 PMC7750391

[IMAG.a.1212-b21] Glasser, M. F., Coalson, T. S., Harms, M. P., Xu, J., Baum, G. L., Autio, J. A., Auerbach, E. J., Greve, D. N., Yacoub, E., Van Essen, D. C., Bock, N. A., & Hayashi, T. (2022). Empirical transmit field bias correction of T1w/T2w myelin maps. NeuroImage, 258, 119360. 10.1016/j.neuroimage.2022.119360 35697132 PMC9483036

[IMAG.a.1212-b22] Gülban, Ö. F., & Huber, R. (2025). Computing geometric layers and columns on continuously improving human (f)MRI data. In J. H. Grafman (Ed.) Encyclopedia of the human brain, second edition (pp. 438–461). Elsevier. 10.1016/B978-0-12-820480-1.00188-1

[IMAG.a.1212-b23] Gulban, O. F., Stirnberg, R., Tse, D. H. Y., Pizzuti, A., Koiso, K., Archila-Melendez, M. E., Huber, L. R., Bollmann, S., Goebel, R., Kay, K., & Ivanov, D. (2026). Whole-brain meso-vein imaging in living humans using fast 7-T MRI. Science Advances, 12(2), eaea4540. 10.1126/sciadv.aea4540 41512066 PMC12787541

[IMAG.a.1212-b25] Henschel, L., Conjeti, S., Estrada, S., Diers, K., Fischl, B., & Reuter, M. (2020). FastSurfer—A fast and accurate deep learning based neuroimaging pipeline. NeuroImage, 219, 117012. 10.1016/j.neuroimage.2020.117012 32526386 PMC7898243

[IMAG.a.1212-b26] Hettwer, M. D., Dorfschmidt, L., Puhlmann, L. M. C., Jacob, L. M., Paquola, C., Bethlehem, R. A. I., Bullmore, E. T., Eickhoff, S. B., & Valk, S. L. (2024). Longitudinal variation in resilient psychosocial functioning is associated with ongoing cortical myelination and functional reorganization during adolescence. Nature Communications, 15(1), 6283. 10.1038/s41467-024-50292-2 PMC11286871 39075054

[IMAG.a.1212-b27] Huber, L., Handwerker, D. A., Jangraw, D. C., Chen, G., Hall, A., Stüber, C., Gonzalez-Castillo, J., Ivanov, D., Marrett, S., Guidi, M., Goense, J., Poser, B. A., & Bandettini, P. A. (2017). High-resolution CBV-fMRI allows mapping of laminar activity and connectivity of cortical input and output in human M1. Neuron, 96(6), 1253–1263.e7. 10.1016/j.neuron.2017.11.005 29224727 PMC5739950

[IMAG.a.1212-b28] Iscan, Z., Jin, T. B., Kendrick, A., Szeglin, B., Lu, H., Trivedi, M., Fava, M., McGrath, P. J., Weissman, M., Kurian, B. T., Adams, P., Weyandt, S., Toups, M., Carmody, T., McInnis, M., Cusin, C., Cooper, C., Oquendo, M. A., Parsey, R. V., & DeLorenzo, C. (2015). Test–retest reliability of freesurfer measurements within and between sites: Effects of visual approval process. Human Brain Mapping, 36(9), 3472–3485. 10.1002/hbm.22856 26033168 PMC4545736

[IMAG.a.1212-b29] Jenkinson, M., Beckmann, C. F., Behrens, T. E., Woolrich, M. W., & Smith, S. M. (2012). Fsl. Neuroimage, 62(2), 782–790. 10.1016/j.neuroimage.2011.09.015 21979382

[IMAG.a.1212-b30] Koopmans, P. J., Barth, M., & Norris, D. G. (2010). Layer-specific BOLD activation in human V1. Human Brain Mapping, 31(9), 1297–1304. 10.1002/hbm.20936 20082333 PMC6870878

[IMAG.a.1212-b31] Küchenhoff, S., Bayrak, Ş., Zsido, R. G., Saberi, A., Bernhardt, B. C., Weis, S., Schaare, H. L., Sacher, J., Eickhoff, S., & Valk, S. L. (2024). Relating sex-bias in human cortical and hippocampal microstructure to sex hormones. Nature Communications, 15(1), 7279. 10.1038/s41467-024-51459-7 PMC11344136 39179555

[IMAG.a.1212-b32] Lee, L. Y., Ziminski, J. J., Frangou, P., Karlaftis, V. M., Wang, Y., Bernhardt, B., Warrier, V., Bethlehem, R. A. I., & Kourtzi, Z. (2025). Neurogenetic phenotypes of learning-dependent plasticity for improved perceptual decisions. Communications Biology, 8(1), 1–11. 10.1038/s42003-025-08212-7 40399642 PMC12095785

[IMAG.a.1212-b33] Lüsebrink, F., Mattern, H., Yakupov, R., Acosta-Cabronero, J., Ashtarayeh, M., Oeltze-Jafra, S., & Speck, O. (2021). Comprehensive ultrahigh resolution whole brain in vivo MRI dataset as a human phantom. Scientific Data, 8(1), 138. 10.1038/s41597-021-00923-w 34035308 PMC8149725

[IMAG.a.1212-b34] Marques, J. P., Kober, T., Krueger, G., van der Zwaag, W., Van de Moortele, P.-F. F., & Gruetter, R. (2010). MP2RAGE, a self bias-field corrected sequence for improved segmentation and T1-mapping at high field. NeuroImage, 49(2), 1271–1281. 10.1016/j.neuroimage.2009.10.002 19819338

[IMAG.a.1212-b35] Mesulam, M.-M. (1998). From sensation to cognition. Brain, 121, 1013–1052. 10.1093/brain/121.6.1013 9648540

[IMAG.a.1212-b36] Morey, R. A., Selgrade, E. S., Wagner II, H. R., Huettel, S. A., Wang, L., & McCarthy, G. (2010). Scan–rescan reliability of subcortical brain volumes derived from automated segmentation. Human Brain Mapping, 31(11), 1751–1762. 10.1002/hbm.20973 20162602 PMC3782252

[IMAG.a.1212-b37] Mugler, J. P., Bao, S., Mulkern, R. V., Guttmann, C. R. G., Robertson, R. L., Jolesz, F. A., & Brookeman, J. R. (2000). Optimized single-slab three-dimensional spin-echo MR imaging of the brain. Radiology, 216(3), 891–899. 10.1148/radiology.216.3.r00au46891 10966728

[IMAG.a.1212-b38] Nerland, S., Jørgensen, K. N., Nordhøy, W., Maximov, I. I., Bugge, R. A. B., Westlye, L. T., Andreassen, O. A., Geier, O. M., & Agartz, I. (2021). Multisite reproducibility and test-retest reliability of the T1w/T2w-ratio: A comparison of processing methods. NeuroImage, 245, 118709. 10.1016/j.neuroimage.2021.118709 34848300

[IMAG.a.1212-b39] Nieuwenhuys, R. (2013). The myeloarchitectonic studies on the human cerebral cortex of the Vogt-Vogt school, and their significance for the interpretation of functional neuroimaging data. Brain Structure & Function, 218(2), 303–352. 10.1007/s00429-012-0460-z 23076375

[IMAG.a.1212-b40] Novek, J., Sprung-Much, T., Nolan, E., & Petrides, M. (2023). Optimal blocking of the cerebral cortex for cytoarchitectonic examination: A neuronavigation-based approach. Cerebral Cortex, 33(6), 2704–2714. 10.1093/cercor/bhac236 35780434

[IMAG.a.1212-b41] Paquola, C., Amunts, K., Evans, A., Smallwood, J., & Bernhardt, B. (2022). Closing the mechanistic gap: The value of microarchitecture in understanding cognitive networks. Trends in Cognitive Sciences, 26(10), 873–886. 10.1016/j.tics.2022.07.001 35909021

[IMAG.a.1212-b42] Paquola, C., Bethlehem, R. A., Seidlitz, J., Wagstyl, K., Romero-Garcia, R., Whitaker, K. J., Vos de Wael, R., Williams, G. B., Vértes, P. E., Margulies, D. S., Bernhardt, B., & Bullmore, E. T. (2019). Shifts in myeloarchitecture characterise adolescent development of cortical gradients. eLife, 8, e50482. 10.7554/eLife.50482 31724948 PMC6855802

[IMAG.a.1212-b43] Paquola, C., Garber, M., Frässle, S., Royer, J., Zhou, Y., Tavakol, S., Rodriguez-Cruces, R., Cabalo, D. G., Valk, S., Eickhoff, S., Margulies, D. S., Evans, A., Amunts, K., Jefferies, E., Smallwood, J., & Bernhardt, B. C. (2025). The architecture of the human default mode network explored through cytoarchitecture, wiring and signal flow. Nature Neuroscience, 28(3), 654–664. 10.1038/s41593-024-01868-0 39875581 PMC11893468

[IMAG.a.1212-b44] Paquola, C., Royer, J., Lewis, L. B., Lepage, C., Glatard, T., Wagstyl, K., DeKraker, J., Toussaint, P.-J., Valk, S. L., Collins, D. L., Khan, A., Amunts, K., Evans, A. C., Dickscheid, T., & Bernhardt, B. C. (2021). The BigBrainWarp toolbox for integration of BigBrain 3D histology with multimodal neuroimaging. eLife, 10, e70119. 10.7554/ELIFE.70119 34431476 PMC8445620

[IMAG.a.1212-b45] Paquola, C., Vos De Wael, R., Wagstyl, K., Bethlehem, R. A. I., Hong, S.-J., Seidlitz, J., Bullmore, E. T., Evans, A. C., Misic, B., Margulies, D. S., Smallwood, J., & Bernhardt, B. C. (2019). Microstructural and functional gradients are increasingly dissociated in transmodal cortices. PLoS Biology, 17(5), e3000284. 10.1371/journal.pbio.3000284 31107870 PMC6544318

[IMAG.a.1212-b46] Park, Y., Namgung, J. Y., Kim, C. Y., Park, Y., & Park, B. (2024). Differences in cortical microstructure according to body mass index in neurologically healthy populations using structural magnetic resonance imaging. Heliyon, 10(12), e33134. 10.1016/j.heliyon.2024.e33134 38984310 PMC11231607

[IMAG.a.1212-b47] Patel, R., Dai, A., Valk, S. L., Desrosiers-Grégoire, G., Devenyi, G. A., & Chakravarty, M. M. (2023). Investigating individual variability in microstructural-functional coupling in the human cortex (p. 2023.05.29.542730). bioRxiv. 10.1101/2023.05.29.542730

[IMAG.a.1212-b48] Polimeni, J. R., Fischl, B., Greve, D. N., & Wald, L. L. (2010). Laminar analysis of 7T BOLD using an imposed spatial activation pattern in human V1. NeuroImage, 52(4), 1334–1346. 10.1016/j.neuroimage.2010.05.005 20460157 PMC3130346

[IMAG.a.1212-b49] Royer, J., Larivière, S., Rodriguez-Cruces, R., Cabalo, D. G., Tavakol, S., Auer, H., Ngo, A., Park, B., Paquola, C., Smallwood, J., Jefferies, E., Caciagli, L., Bernasconi, A., Bernasconi, N., Frauscher, B., & Bernhardt, B. C. (2023). Cortical microstructural gradients capture memory network reorganization in temporal lobe epilepsy. Brain, 146(9), 3923–3937. 10.1093/brain/awad125 37082950 PMC10473569

[IMAG.a.1212-b50] Royer, J., Rodríguez-Cruces, R., Tavakol, S., Larivière, S., Herholz, P., Li, Q., Vos de Wael, R., Paquola, C., Benkarim, O., Park, B.-Y., Lowe, A. J., Margulies, D., Smallwood, J., Bernasconi, A., Bernasconi, N., Frauscher, B., & Bernhardt, B. C. (2022). An open MRI dataset for multiscale neuroscience. Scientific Data, 9(1), 569. 10.1038/s41597-022-01682-y 36109562 PMC9477866

[IMAG.a.1212-b51] Sanides, F. (1962). Die Architektonik des menschlichen Stirnhirns zugleich eine Darstellung der Prinzipien seiner Gestaltung als Spiegel der stammgeschichtlichen Differenzierung der Grosshirnrinde. 201. http://catalog.hathitrust.org/api/volumes/oclc/6684303.html

[IMAG.a.1212-b52] Shams, Z., Norris, D. G., & Marques, J. P. (2019). A comparison of in vivo MRI based cortical myelin mapping using T1w/T2w and R1 mapping at 3T. PLoS One, 14(7), e0218089. 10.1371/journal.pone.0218089 31269041 PMC6609014

[IMAG.a.1212-b54] Sprooten, E., O’Halloran, R., Dinse, J., Lee, W. H., Moser, D. A., Doucet, G. E., Goodman, M., Krinsky, H., Paulino, A., Rasgon, A., Leibu, E., Balchandani, P., Inglese, M., & Frangou, S. (2019). Depth-dependent intracortical myelin organization in the living human brain determined by in vivo ultra-high field magnetic resonance imaging. NeuroImage, 185, 27–34. 10.1016/j.neuroimage.2018.10.023 30312809 PMC6289812

[IMAG.a.1212-b55] Stüber, C., Morawski, M., Schäfer, A., Labadie, C., Wähnert, M., Leuze, C., Streicher, M., Barapatre, N., Reimann, K., Geyer, S., Spemann, D., & Turner, R. (2014). Myelin and iron concentration in the human brain: A quantitative study of MRI contrast. NeuroImage, 93(P1), 95–106. 10.1016/j.neuroimage.2014.02.026 24607447

[IMAG.a.1212-b56] Sui, Y. V., Masurkar, A. V., Rusinek, H., Reisberg, B., & Lazar, M. (2022). Cortical myelin profile variations in healthy aging brain: A T1w/T2w ratio study. NeuroImage, 264, 119743. 10.1016/j.neuroimage.2022.119743 36368498 PMC9904172

[IMAG.a.1212-b57] Sydnor, V. J., Petrie, D., McKeon, S. D., Famalette, A., Foran, W., Calabro, F. J., & Luna, B. (2025). Heterochronous laminar maturation in the human prefrontal cortex (p. 2025.01.30.635751). bioRxiv. 10.1101/2025.01.30.635751

[IMAG.a.1212-b58] Valk, S. L., Kanske, P., Park, B., Hong, S.-J., Böckler, A., Trautwein, F.-M., Bernhardt, B. C., & Singer, T. (2023). Functional and microstructural plasticity following social and interoceptive mental training. eLife, 12, e85188. 10.7554/eLife.85188 37417306 PMC10414971

[IMAG.a.1212-b59] Von Economo, C., & Koskinas, G. (1925). Die Cytoarchitektonik der Hirnrinde des erwachsenen Menschen. Springer. 10.1001/archneurpsyc.1926.02200300136013

[IMAG.a.1212-b60] Waehnert, M. D., Dinse, J., Weiss, M., Streicher, M. N., Waehnert, P., Geyer, S., Turner, R., & Bazin, P.-L. (2014). Anatomically motivated modeling of cortical laminae. NeuroImage, 93, 210–220. 10.1016/J.NEUROIMAGE.2013.03.078 23603284

[IMAG.a.1212-b62] Wei, W., Yin, Y., Zhang, Y., Li, X., Li, M., Guo, W., Wang, Q., Deng, W., Ma, X., Zhao, L., Palaniyappan, L., & Li, T. (2022). Structural covariance of depth-dependent intracortical myelination in the human brain and its application to drug-naïve schizophrenia: A T1w/T2w MRI study. Cerebral Cortex, 32(11), 2373–2384. 10.1093/cercor/bhab337 34581399

[IMAG.a.1212-b63] Weiskopf, N., Edwards, L. J., Helms, G., Mohammadi, S., & Kirilina, E. (2021). Quantitative magnetic resonance imaging of brain anatomy and in vivo histology. Nature Reviews Physics, 3(8), 570–588. 10.1038/s42254-021-00326-1

[IMAG.a.1212-b64] Weiskopf, N., Suckling, J., Williams, G., Correia, M. M., Inkster, B., Tait, R., Ooi, C., Bullmore, E. T., & Lutti, A. (2013). Quantitative multi-parameter mapping of R1, PD(*), MT, and R2(*) at 3T: A multi-center validation. Frontiers in Neuroscience, 7, 95. 10.3389/fnins.2013.00095 23772204 PMC3677134

[IMAG.a.1212-b65] Whitaker, K. J., Vértes, P. E., Romero-Garcia, R., Váša, F., Moutoussis, M., Prabhu, G., Weiskopf, N., Callaghan, M. F., Wagstyl, K., Rittman, T., Tait, R., Ooi, C., Suckling, J., Inkster, B., Fonagy, P., Dolan, R. J., Jones, P. B., Goodyer, I. M., & Bullmore, E. T. (2016). Adolescence is associated with genomically patterned consolidation of the hubs of the human brain connectome. Proceedings of the National Academy of Sciences, 113(32), 9105–9110. 10.1073/pnas.1601745113 PMC4987797 27457931

